# Education Research: Bridging the Artificial Intelligence Training Gap: Evidence from a National Survey of Italian Neurology Residents

**DOI:** 10.1212/NE9.0000000000200289

**Published:** 2026-01-27

**Authors:** Claudio Vozzi, Matteo Sibilla, Daniel Sandri, Valentina Marinato, Giulia Micolonghi, Serena Oliveri, Massimo Filippi, Sara Marceglia, Alberto Priori

**Affiliations:** 1 Clinical Neurology Unit, “Azienda Socio-Sanitaria Territoriale Santi Paolo e Carlo”, University of Milan, Italy;; 2 “Aldo Ravelli” Center for Neurotechnology and Experimental Brain Therapeutics, Department of Health Sciences, University of Milan, Italy;; 3 Neurology Unit, IRCCS San Raffaele Scientific Institute, Milan, Italy;; 4 Vita-Salute San Raffaele University, Milan, Italy; and; 5 Clinical Neurology Unit, “Azienda Socio-Sanitaria Territoriale Santi Paolo e Carlo”, “Aldo Ravelli” Center for Neurotechnology and Experimental Brain Therapeutics, Department of Health Sciences, University of Milan, Italy.

## Abstract

**Background and Objectives:**

As artificial intelligence (AI) rapidly becomes an integral tool in clinical neurology, future clinicians will need to master its application in patient care. While previous studies focused primarily on medical students' perspectives, our survey, addressed to Italian neurology residents, aims to assess their familiarity with AI tools and identify educational needs of learners close to clinical practice and care delivery.

**Methods:**

A cross-sectional, web-based survey designed by the University of Milan was distributed nationwide to neurology residents from May 22 through July 30, 2025. The questionnaire included items on demographics, self-assessed AI knowledge, exposure to AI training, clinical applications, perceived challenges, and attitudes toward the impact of AI on neurology practice. Descriptive statistics and association analyses were performed.

**Results:**

A total of 173 residents (12.7%) completed the survey, with 37% affiliated with research hospitals. Although AI is frequently used in practice, with 40.7% using AI tools at least on a weekly basis, only 30.7% rated their knowledge as “good” or higher. Paradoxically, most residents (79.2%) reported no curriculum-integrated AI education, which was instead significantly associated with geographical background (Χ^2^ = 45.36, *p* < 0.001), while nearly all respondents expressed interest in further AI training (93.1%). The most familiar application was generative AI for clinical decision support (64.2%), and neuroimaging was rated as the first AI integration area (mean priority 2.99). Main barriers to AI application included concerns about reliability (71.1%) and data privacy (43.9%). Regarding career perspectives, 41.6% of residents believed that AI will create new job opportunities, whereas most (89%) agreed that the AI revolution will not replace human professionals.

**Discussion:**

Despite the limited number of participants, our survey provides a representative snapshot of AI knowledge, use, and attitudes among neurology residents in Italy, a critical country in the digital health care transformation, contributing important evidence for international shared recommendations on AI educational needs to support future clinical practice. Although AI tools are widely used, most residents had only basic knowledge and limited formal training, prompting calls for more structured, learner-centered educational modules to be integrated into neurology curricula.

## Introduction

Artificial intelligence (AI) is enabling profound advancements across various fields of medicine, revolutionizing diagnostic processes, prognostic modeling, and therapeutic strategies. In this setting, neurology is no exception.^1,2^ AI is an umbrella term used to define different technologies, spanning from machine learning to deep learning and extending to more complex cognitive computing systems and widespread large language models (LLMs). Collectively, these techniques have shown promising results in automatic neuroimage analysis, clinical decision support, and patient monitoring in neurologic settings.^3,4^ In particular, LLMs such as ChatGPT, Gemini, and Perplexity can be trained for text classification in different clinical contexts (e.g., medical records, patient histories, or clinical trial reports) to automatically extract relevant features as well as generate diagnostic hypotheses and predict treatment outcomes.^5^ However, despite their potential accuracy, answers provided by such systems are still outperformed by neurologists' decisions in real-world scenarios that require experience-trained clinical acumen.^6^ Moreover, advanced machine-learning applications can expand the neurologist's armamentarium by enabling the identification of biomarkers for outcome prediction, facilitating early risk stratification and personalized therapy, and providing deeper insights into the underlying pathogenetic mechanisms. These technologies have the potential to address virtually the whole spectrum of neurologic diseases.^7,8^

According to Stanford AI Index 2025, Italy currently holds a mid-level position internationally in AI adoption, gradually advancing strategic policies and developing future investment plans to support AI implementation, which includes the creation of dedicated degree courses by universities to fill this gap.^9,10^ Italy's involvement in developing LLMs and participation in AI-driven medical research align with the broader trend of expanding clinical AI applications, positioning Italy as an emerging contributor to the international AI scene in medicine.^11^

Conversely, medical education programs are struggling to keep up with the rapid evolution of these technologies, facing the challenge of balancing innovation and established knowledge, in a setting where the volume of available medical knowledge surpasses what learners can realistically acquire and retain. Therefore, the current issue is to promote educational classes on AI without placing an additional burden on the educational pathway, which is already extensive and demanding.^12^ At the same time, clear policies and regulations are needed to guide educational institutions, as they face the challenge of appropriately adapting curricula, teaching strategies, and assessment methods to meaningfully integrate AI tools.^13^ Indeed, the next generation of clinicians is now required to master not only clinical expertise but also a profound understanding of AI principles, limitations, and ethical implications.^14^

Despite this, several studies report a lack of structured AI training in medical curricula, with learners expressing uncertainty about being properly trained to use AI in practice.^15,16^ Although some leading US institutions have begun introducing AI teaching modules into medical training, these initiatives remain the exception rather than the rule, with many countries outside the United States, especially in Europe and low and middle-income regions, still facing gaps in AI training despite its potential benefits for overcoming health care disparities.^17,18^ The educational AI training gap is particularly relevant in neurology, where instrumental testing such as neuroimaging and EEG interpretation plays a decisive role in diagnosis and outcome prediction, and AI is expected to enhance the accuracy and efficiency of interpretation.^19^

Some recent literature recommends conducting national surveys among medical students to assess their attitudes and expectations regarding AI education, serving as a guide to curriculum development by identifying realistic goals for future physicians, clarifying expectations, and outlining the resources and knowledge faculty will need.^20^ Previous surveys have mainly explored medical students' perspectives on AI in their professional future, focusing on their familiarity with AI platforms, inclusion of AI classes in curricula, and concerns about the impact on their professional careers.^21,22^

We are currently in a time of rapid adoption and experimentation with AI in medicine. During this critical phase, obtaining input from learners is essential. Neurology residents hold a unique position, serving simultaneously as learners and educators, clinicians delivering patient care, and firsthand observers of clinical workflows. Their perspectives are invaluable for guiding the integration of AI into both training programs and clinical practice. The objective of our survey was to specifically examine Italian neurology residents' current use of AI tools and perceived educational gaps, as well as unexpressed educational needs, within neurology residency training.

## Methods

### Participants

A cross-sectional, web-based survey was developed by the University of Milan and distributed to neurology residents at centers across the country from May 22 to July 30, 2025, through institutional mailing lists and professional networks. A sample size of 147 participants was required to ensure an 80% confidence level with a 5% margin of error, increasing to 181 participants for an 85% confidence at the same margin. All questionnaires were kept anonymous, and the data collected did not allow the recognition of individuals (details in Research Ethics).

### Questionnaire Development

The questionnaire was developed as part of an initiative arising from a preliminary meeting among neurology residents from the University of Milan, aimed at assessing training needs and related issues. The first draft was designed by 5 residents (C.V., M.S., D.S., V.M., G.M.), taking inspiration from models described in the literature,^21,22^ and was preliminarily distributed to neurology residents at San Paolo Hospital, who provided valuable feedback that was subsequently incorporated into the final version. Then, the integrated version was reviewed by a neuropsychology expert in preference studies (S.O.), as well as a biomedical engineer expert in AI (S.M.) and 2 Neurology Specialty School Directors (A.P. and M.F.). The questionnaire was developed in Italian and administered online through Google Forms.

The survey consisted of 25 multiple-choice questions and was built so that residents could only select 1 answer, unless otherwise instructed; 23 questions were mandatory while 1 open-ended question was also included, requiring respondents to provide written answers. Finally, 1 question was asked only to responders who chose a specific answer to the previous one, thus making it not mandatory. The questionnaire was structured into 3 main sections to investigate, respectively, the *demographics* of participants; respondents' background *knowledge, training, and perception* toward AI; and residents' *experience, attitudes, and perspectives* of AI tools in clinical practice. This structure was intended to offer a comprehensive insight into the interplay between educational background, institutional environment, and attitudes toward AI among neurology residents. The full questionnaire is presented in eFigure 1.

### Demographics

Participants were first asked to provide key basic nonidentifying demographic information (name, sex, and age were omitted), such as their current year of residency, the geographical area of their university affiliation, and the type of hospital facility where they were training. These hospital facilities are categorized into purely clinical facilities with varying inpatient capacity and research hospitals with public funding—the “Istituti di Ricovero e Cura a Carattere Scientifico” (IRCCS in Italian, *Scientific Institute for Research, Hospitalization and Healthcare*). This section included 3 mandatory questions.

### AI Knowledge, Training, and Perceptions

The second section focused on both self-perceived and actual knowledge of AI among residents. Participants were first invited to rate their self-perceived knowledge of the topic and then were asked about their previous or planned exposure to AI training (including academic courses or extracurricular modules) and the AI tools they were most familiar with in clinical practice. Furthermore, they were requested to rank by priority the most suitable areas for AI integration in neurology and to identify the main barriers to its application. The final question addressed the environmental impact of AI tools and their perceived relevance. This section included 7 mandatory questions and 1 optional question.

### AI Experience, Interaction, and Perspectives

The final section focused on the practical and attitudinal aspects of AI in clinical neurology. Residents reported on the frequency and context in which they used AI tools, their modes of interaction, and the perceived consistency and reliability of answers provided by AI platforms compared with traditional sources of education (tutors and textbooks). Finally, participants were asked to reflect on the broader impact of AI on neurologic practice, including its potential influence on career opportunities and the perceived need for further education and training in this rapidly evolving field. This section included 13 mandatory questions and 1 optional question.

### Data Analysis

Descriptive statistics were used to analyze the results: for each question, the percentage of responses was gathered to analyze the distribution of answers. Then, secondary association analyses were performed. In particular, demographics were compared with levels of self-rated AI knowledge and exposure to AI training courses, to better disclose associations with the level of educational maturity, the geographical location, and the type of working environments. A further analysis was conducted comparing the hospital facilities of the residents and the perceived inconsistency between AI-generated answers in clinical scenarios and answers provided by the tutors.

To do so, frequencies of answers were compared across different groups using the Pearson χ^2^ test (using JASP open-source software version 0.19.3), with a *p* value of <0.05 regarded as statistically significant. To further explore specific associations, when the overall χ^2^ test was statistically significant, we re-aggregated variables into dichotomous categories (i.e., “exposure to AI training” vs “not exposure to AI training” and “IRCCS hospitals” vs “other facilities”), using the same statistical tests.

### Research Ethics

Participant anonymity was fully disclosed before the start of the survey and was strictly guaranteed: no personally identifiable information (such as names, email addresses, or institutional affiliations) was collected. The Ethical Committee of the University of Milan (*Comitato Etico dell’Università degli Studi di Milano*) was consulted as the Institutional Review Board (IRB) for this study. Given that the questionnaire was included as an optional curricular activity approved by the board of the School of Neurology involving an anonymous survey administered to medical residents and considering that data were collected in a completely anonymized manner, formal ethical approval and informed consent were deemed unnecessary.

### Data Availability

Anonymized data not published within this article will be made available by request from any qualified investigator. Data are displayed in eFigure 2.

## Results

A total of 173 neurology residents completed the survey, accounting for 12.7% of the estimated total number of neurology residents in Italy (n = 1,359), based on data provided by the Italian Ministry of University and Research.^23^ Based on the SurveyMonkey sample size calculator, our sample size of 173 provides a 5% margin of error and a confidence level between 80% and 85%.^24^

Almost half of the responses came from Northern Italy (43.4%; n = 75), with 13 respondents from the Italian islands (7.5%); the remaining answers were fairly evenly distributed between Central and Southern Italy. The Italian neurology residency program consists of 4 years of training: residency years were evenly represented in the responses, each accounting for approximately 1 quarter (eFigure 2). Concerning the working environment, 37% (n = 64) of residents trained primarily in a research hospital (IRCCS) and almost the same proportion trained in inpatient departments with fewer than 20 beds (40.5%; n = 70).

Regarding AI knowledge, nearly two-thirds of respondents (63%; n = 109) rated their knowledge about AI as “elementary” while 6.4% (n = 11) reported no knowledge at all. Among the remaining respondents, 27.2% (n = 47) rated their knowledge as “good” and 3.5% (n = 6) as “advanced.” Moreover, two-thirds of residents (79.2%; n = 137) reported not having received any training on medical applications of AI, and among them, 68.8% (n = 117) indicated that their curriculum includes no AI training. Only 12.1% (n = 21) of residents were aware of curricular training on AI in medical care, whereas 19.1% (n = 33) of learners had received AI-in-medicine training through extracurricular courses, such as seminars.

### AI Knowledge, Training, and Perceptions

**Figure 1:**
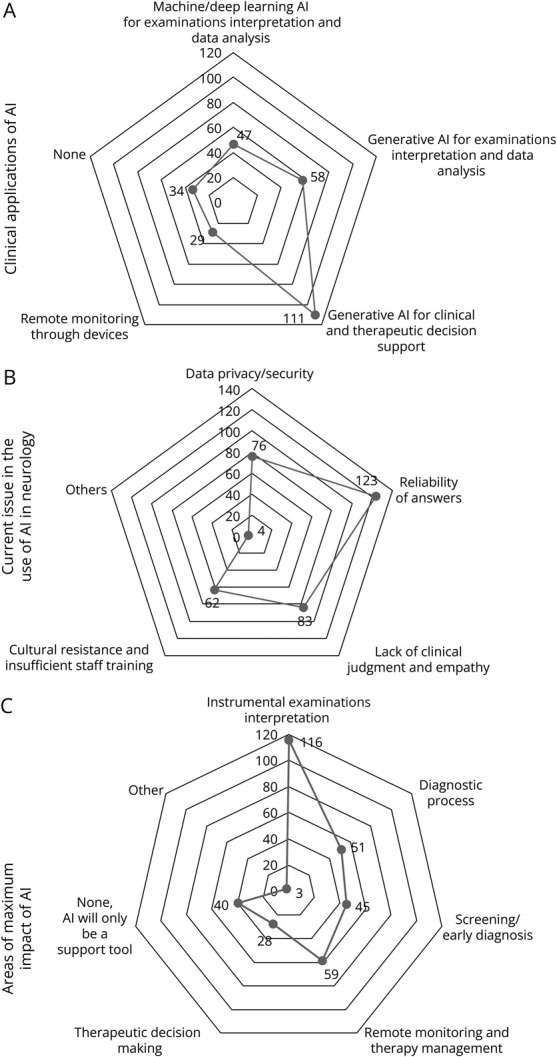
Clinical Applications of AI in Neurology and Concerns About Their Use Radar charts were used to visualize answer distribution. (A) The radar chart visualizes the distribution of most common AI applications in their clinical practice among residents, each having selected 1 among 5 possible options. Each axis represents a distinct AI application, with values indicating the count of residents, in terms of the absolute number (N) who chose that option. (B) The radar chart visualizes the distribution of the most common perceived barriers and limitations of AI among residents, each able to choose more than 1 answer (multiple responses allowed). Each axis represents a distinct AI concern, with values indicating the count of residents, in terms of the absolute number (N) who chose that option. (C) The radar chart visualizes the distribution of the most suitable areas where residents perceive AI could replace neurologists, with each participant allowed to select multiple responses. Each axis represents a distinct AI concern, with values indicating the count of residents, in terms of the absolute number (N) who chose that option.

As shown in Figure 1A, when asked about the most common clinical application of AI, 64.2% (n = 111) of respondents knew about or had used generative AI platforms (i.e., ChatGPT, Perplexity, and Meta) for clinical decisional and therapeutic support and 33.5% (n = 58) reported using generative AI platforms for neuroradiologic or neurophysiologic examination interpretation, while 27.2% (n = 47) were familiar with machine learning and deep learning algorithms for automated interpretation of diagnostic testing. Finally, 19.7% (n = 34) had never used or did not know about any of those AI applications.

As shown in Figure 1B, questions about limits of AI were administered: most of the residents (71.1%, n = 123) expressed concerns about the reliability of AI outputs while a substantial portion (43.9%, n = 76) were concerned about privacy and data security. Moreover, 39.9% of participants (n = 69) believe that the environmental impact of AI should be prioritized before its application while 46.8% (n = 81) consider it a critical issue but do not view it as always a priority. A question was asked about ranking by priority (1–6 from the most to least suitable) 6 areas of neurology in which AI integration would have the greatest impact. The area of neuroradiology was the highest ranked, with a mean position of 2.99, and it was the most voted for both first (n = 49) and second (n = 37) positions, thus indicating a clear trend in the answers. The remaining categories, that is, diagnostic workup (including anamnesis and physical examinations), personalized treatment, remote monitoring, and clinical research, exhibited similar ranking results.

As shown in Figure 1C, residents reported concerns about AI taking over neuroradiology image interpretation (67.1%, n = 116), remote treatment response monitoring (34.1%, n = 59), and diagnostic workup (29.5%, n = 51).

### AI Experience, Interaction, and Perspectives

**Figure 2:**
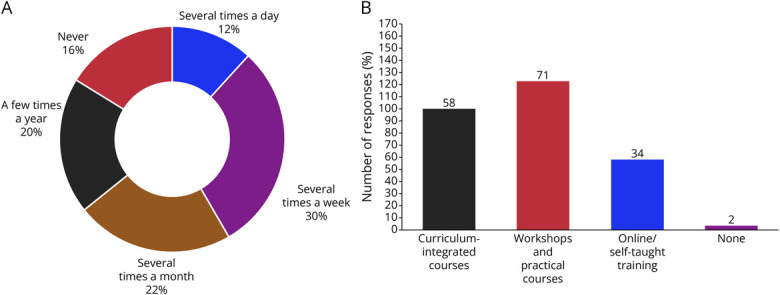
Frequency of AI Tool Application in Clinical Practice and Preferred Training Methods (A) The doughnut chart shows the response percentages (relative to the total number of residents) for each frequency of AI tool usage. Only 1 answer was allowed. (B) The graph shows the results of preferred AI teaching methods by residents. On the x-axis, each answer option is represented while on the y-axis, the absolute number (N) of responses given by residents is shown. Multiple choices were allowed. The percentages reported were calculated relative to the total number of respondents (not the total number of responses).

Figure 2A illustrates the frequency of AI tool use in clinical practice, with a significant portion of residents (40.7%, n = 72) reporting at least weekly use, comprising 30.1% using them several times a week and 11.6% several times a day. Only 16.2% (n = 28) reported no use at all. Regarding how frequently residents prefer AI platforms as a source of clinical information over traditional resources, 87 residents (50.3%) reported “often” or “always” using AI instead of textbooks while 70 (40.5%) reported doing so “rarely” or “not at all.” Some residents reported that they frequently compare clinical information obtained from traditional sources with that from AI (37.6%, n = 65), but only 27.7% (n = 18) found the answers to be generally better and more detailed than those of professors.

Regarding reliability of AI-generated answers, 39.3% of residents (n = 68) reported noninferiority of AI answers when compared with those provided by tutors or professors; still, a relevant number of respondents (32.9%; n = 57) found AI answers to be less consistent than their tutors' answers. Although nearly two-thirds of residents (67.6%, n = 117) perceive that they “often” or “always” need to check for the consistency of AI answers, most of them (87.3%, n = 151) reported being positive about the integration of AI into clinical practice. Of interest, almost unanimously (93.1%, n = 161), the residents answered positively when asked whether they would be open to attending an educational module on how to best use AI in clinical neurologic practice, thereby giving a clear direction to the category's intent.

Figure 2B shows the results for the desirable teaching method. Most (71.1%, n = 123) expressed a preference for practical workshops, 57.8% (n = 100) preferred curriculum-integrated courses, and one-third (n = 58) voted for self-teaching material. Only 3 residents reported no need for AI training in neurology residency. However, 23.1% (n = 40) of residents believe that AI will only be a support to clinical practice and never a substitute. Accordingly, 89% (n = 154) of respondents mostly or completely agree with the statement that “AI will revolutionize the neurologist's job but will never completely replace it,” and 41.6% of responders (n = 71) feel that AI might bring more career opportunities and economic growth to the category in the near future.

### Associations Among Responses

Secondary association analyses using the Pearson χ^2^ test were performed: self-rated knowledge did not significantly differ across the years of residency (Χ^2^ = 4.60, df = 9, *p* = 0.87), the geographical origin of the affiliated university hospital (Χ^2^ = 15.00, df = 9, *p* = 0.09), or the type of hospital facility (Χ^2^ = 13.18, df = 9, *p* = 0.16). However, when aggregating AI knowledge into tech-savvy (categories “advanced” and “good”) vs non–tech-savvy (categories “basic” and “no knowledge”), there was a borderline significant association with geographical origin (Χ^2^ = 7.65, df = 3, *p* = 0.05), with the most notable difference among responders from Southern Italy who rated themselves as tech-savvy (Pearson-adjusted standardized residuals +1.71). No significant association was found between the modality of exposure to AI training and the perceived AI knowledge (Χ^2^ = 20.37, df = 12, *p* = 0.06).

Significant associations instead emerged between the modality of exposure to AI training and the demographic variables, respectively, with the year of residency (Χ^2^ = 22.33, df = 12, *p* = 0.03), the geographical location of affiliated university hospital (Χ^2^ = 45.36, df = 12, *p* < 0.001), and the type of hospital facility (Χ^2^ = 23.51, df = 12, *p* = 0.02). Specifically, the most consistent deviations from expected frequencies in the χ^2^ analysis, as indicated by the Pearson-adjusted residuals, were observed for first-year residents selecting “No, but it is planned later in the curriculum” (+3.96), residents from Southern Italy selecting “Yes, through extracurricular courses or specialist seminars” (+5.41), and residents at medium-sized general hospitals selecting “No, and it is not planned” (+3.05).

To confirm these findings, exposure to AI training was aggregated into dichotomous variables (“no exposure” vs “exposure”) and hospital types into “IRCCS hospitals” vs “other hospitals.” This classification may facilitate understanding of whether the type of facility (research vs nonresearch hospitals) constitutes a critical determinant of residents' exposure to AI training and associated opportunities. A significant association remained only between the geographical origin and the AI training exposure (Χ^2^ = 24.43, df = 3, *p* < 0.001), with Southern Italy residents reporting exposure during their training showing the strongest association (Pearson-adjusted residuals +3.81). Finally, discrepancies between AI-generated answers and tutors' answers were independent of hospital facility type (Χ^2^ = 11.77, df = 12, *p* = 0.47).

**Figure 3:**
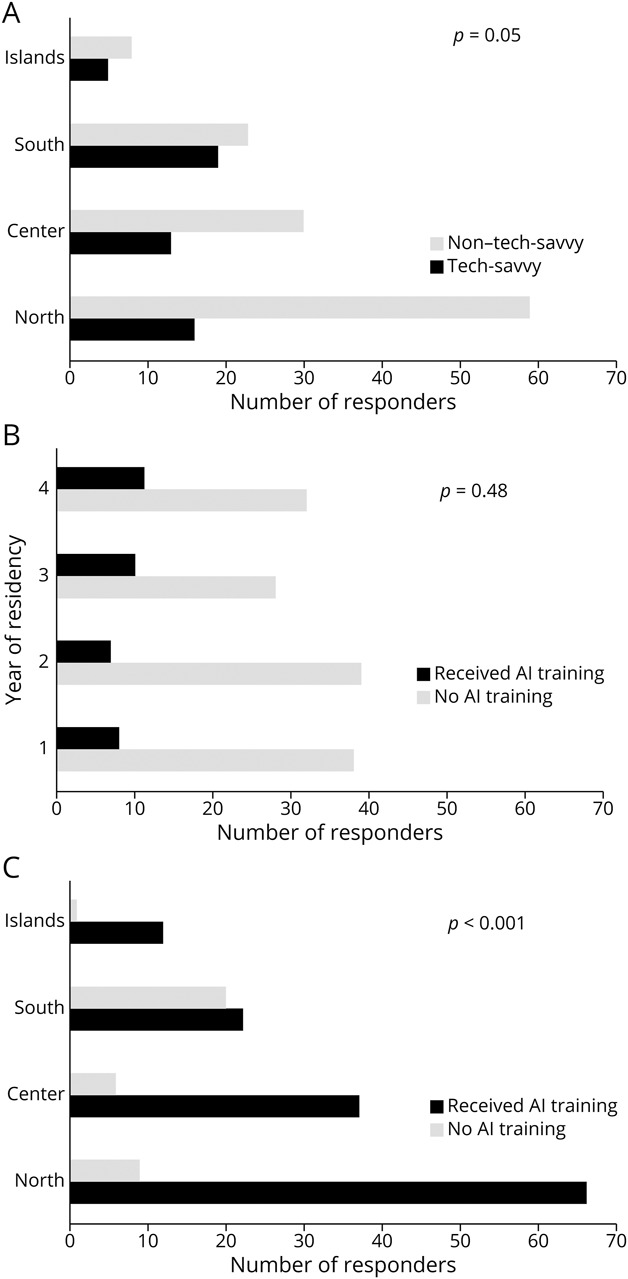
Association Analyses Between the Demographics and the Self-Rated AI Knowledge and Training On the x-axis, the absolute number (N) of responders is given; on the y-axis, the demographic categorical variables are provided. Pearson chi-square tests were performed, and *p* values were reported for each analysis. (A) Analysis of association between the respondent's geographical origin of university affiliation and the self-rated AI knowledge. Residents' answers to the question about self-rated AI knowledge were reclassified into 2 categories: “tech-savvy” vs “non–tech-savvy” (details in Methods). (B) Analysis of association between the respondents' year of residency and the exposure to AI training. Residents' answers regarding exposure to AI training were reclassified into 2 categories: “exposure to AI training” vs “no exposure to AI training” (details in Methods). (C) Analysis of association between the respondent's geographical origin of university affiliation and the exposure to AI training. Residents' answers regarding exposure to AI training were reclassified into 2 categories: “exposure to AI training” vs “no exposure to AI training” (details in Methods).

Figure 3 illustrates the most relevant results from association analyses.

## Discussion

Our national survey provides a comprehensive assessment of AI knowledge, use, and educational needs among neurology residents in Italy, a country actively striving to overcome the gap between the educational potential of AI and its actual clinical implementation.^25^ Italy's strong integration in global neurologic research networks and active participation in European standardization efforts make these results relevant beyond national borders. Italy, currently holding an intermediate position in AI adoption but demonstrating substantial potential through significant investment in this field, may serve as a representative model for a wide range of countries with comparable levels of adoption and prospects. In addition, neurology residents hold the unique position of being both learners and health care providers, thus providing a practice-oriented educational perspective. Investigating and highlighting their educational needs is, therefore, paradigmatic for developing international consensus-driven recommendations on safe and effective AI curricular integration.

Although AI is already frequently used in practice—with most residents using AI tools at least on a weekly basis and most of them relying on free demo platforms—only a minority rated their knowledge as at least “good,” which aligns with trends reported in large-scale surveys among medical students. The use of generative AI platforms for diagnostic and therapeutic support was the most prevalent, highlighting the widespread diffusion of open LLMs as assistive technologies in real-life clinical scenarios. The prioritization of neuroimaging as a key area for AI integration in clinical practice is consistent with the literature, which identifies radiology at the forefront of addressing concerns and pioneering surveys about AI implementation in medicine.^26,27^ Paradoxically, most residents reported no curriculum-integrated AI education while nearly all respondents expressed interest in further training on the topic, with a slight preference for workshops and practical courses over formal educational classes.

This is consistent with international reports of worldwide learners' demand for inclusion of AI training in their academic path.^28^ Of interest, the level of AI knowledge and the exposure to AI training were consistent, regardless of the advancement in residency and the type of hospital facility, whereas the association with geographical background could be attributed to participation bias; this may reflect the greater adhesion of tech-savvy respondents from Southern Italy, where the questionnaire was distributed at a later time point. Overall, these findings confirm the significant gap between the widespread use of AI tools and the limited formal education received by residents, in line with international reports showing that, despite increasing exposure to AI in clinical settings, structured training is still lacking and claimed by residents.^29^

Main barriers to AI application included concerns about the reliability of the answers and data privacy/security, which remain significant limitations to broader adoption.^30^ Moreover, less than half of the residents reported noninferiority of AI answers when compared with those provided by tutors or professors, regardless of affiliation or type of hospital facility. Regarding future career impact, the largest proportion of residents believed that AI will create new job opportunities, whereas most agreed that the AI revolution will not replace human professionals. Such a perception—that AI will support rather than replace clinicians—is widely supported in the literature.^31^

Despite having acceptable statistical power, the number of respondents to our survey accounted only for 12.7% of the total sample size, thus potentially producing a selection bias, favoring those more interested in AI. Alongside this, the online distribution may have potentially contributed to this, especially toward tech-savvy residents, which might also partly explain some of our geographically associated results. Furthermore, the self-evaluation nature of our questions is less ideal for obtaining reproducible knowledge assessment results, and no evaluation on the quality of AI training was conducted for respondents who stated having had any. Finally, the rapidly evolving landscape—including AI training as well—may render our results temporary, underlining the need for future follow-up evaluations on the topic.

Results obtained from this work demonstrate a gap between the frequent use of AI platforms by residents and AI curricular training exposure. As other studies suggested for undergraduate medical programs, a staged model could be adapted for residency training as well.^32^ A recent European review proposes categorizing AI competency into 3 progressive levels (basic awareness, practical application, and advanced expertise) and explores how these competencies can be integrated across undergraduate, postgraduate, and continuing professional development stages in medical education.^33^ Educational strategies should, therefore, focus on empowering residents to critically appraise and effectively collaborate with AI tools, rather than perceiving AI as a threat to their professional roles.

Building on the results of our survey, we suggest alternative educational approaches to be integrated during residency, such as seminars addressing the technical bases of AI and ethical issues highlighted in the literature. For the latter, our respondents were particularly concerned about patient confidentiality, algorithmic bias, and informed consent.^34^ Furthermore, given the current limited use of institutional AI platforms among residents, expanding access to these resources could substantially enhance training opportunities and clinical practice integration. As shown by our survey, neuroimaging stands out as the most promising field for AI integration in neurology: this field could benefit greatly from AI tools such as advanced imaging analysis, automated lesion detection, and decision support systems. To boost residents' competencies, education programs can adopt AI-powered interactive platforms for real-time image interpretation and feedback, alongside simulation-based modules exposing trainees to rare or complex neuroimaging cases. In such a context, simulation-based education has shown promise in enhancing residents' skills and confidence with AI-augmented tools, serving as a valuable complement to traditional teaching methods.^35,36^

Furthermore, interdisciplinary sessions involving other professionals, such as biomedical engineers and data scientists, should be suggested and structured as seminars and practical workshops, with a basic teaching of computer science and neural networks for a better understanding of what AI can do for other sciences, how to use it for a comprehensive mathematical understanding of data, and how to use it for a better interpretation of correlation among clinical variables. These sessions would also provide opportunities to assess residents' skills through real-case scenarios that challenge AI-driven platforms.^37^

For example, well-documented biases of open LLMs, such as their tendency to memorize answers and extract information from unauthenticated sources, could be leveraged as a teaching tool to encourage residents' active participation in providing input and training institutionally provided AI platforms.^38^ Finally, universities should encourage residents not only to pursue theses or research projects using AI methods to investigate neurologic diseases, biomarkers, or neuroimaging data but also to take initiative in conducting internal surveys aimed at assessing the achievement of educational goals, providing faculties with valuable feedback to support continuous improvement of the curriculum. Ultimately, coordinating single-center initiatives and data collection on a national and international scale is essential to develop and rigorously validate a balanced, consensus-driven teaching framework, which can then be proposed for widespread adoption by medical institutions worldwide.

**Figure 4:**
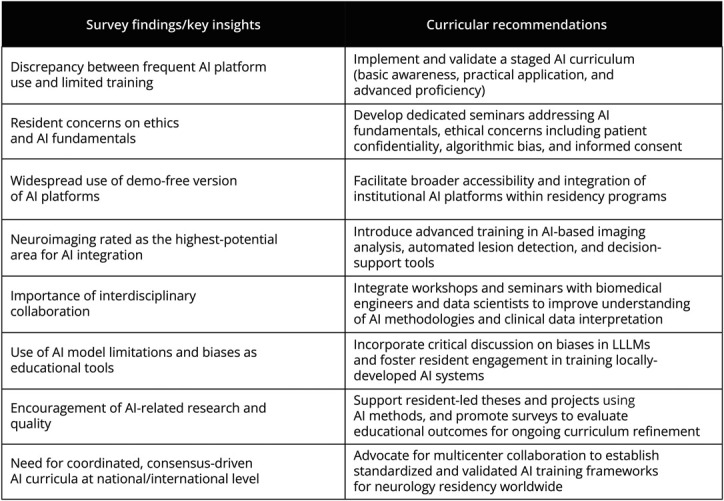
The Table Summarizes the Major Survey Findings and Key Insights Alongside Corresponding Curricular Recommendations Aimed at Enhancing Artificial Intelligence Competencies in Neurology Residency Training

Figure 4 illustrates the key points discussed, providing a clear synthesis of our main curricular proposals.

Our results underscore the urgent need to integrate comprehensive AI curricula into neurology residency programs, as such curricula should cover not only technical aspects but also ethical, legal, and social implications of AI in clinical practice.^39^ Currently, existing guidelines address broader AI applications across medical and health care specialties, while specific efforts to develop dedicated frameworks in neurology—especially for research purposes—have recently been initiated.^40,41^ Notably, our findings confirm widespread use of AI tools, a strong demand for structured education, and significant concerns about reliability and privacy.

Given the current developments, sharing these results now is particularly relevant for informing future directions. As proficiency with these technologies becomes a professional expectation, the next generation of neurologists might be called on to answer for not using AI platforms in the event of diagnostic omission and/or treatment errors. Nowadays, it is essential to integrate robust AI training into residency programs, ensuring that the future generation of neurologists is prepared to harness the full potential of these new technologies in their clinical practice.

In conclusion, our study highlights the pivotal role of residents in shaping the future incorporation of AI applications in neurology during this ongoing phase of AI integration in health care. Positioned at the intersection of education and clinical care, residents offer critical insights as learners, teachers, and frontline clinicians. Their feedback is, therefore, crucial to ensure that AI integration aligns with practical clinical needs and educational goals, ultimately supporting safe, effective, and meaningful adoption within neurology training and practice.

## Glossary

AI: artificial intelligence
IRCCS: Scientific Institute for Research, Hospitalization and Healthcare
LLMs: large language models

## References

[1] Esteva A, Robicquet A, Ramsundar B, et al. A guide to deep learning in healthcare. Nat Med. 2019;25(1):24-29. doi:10.1038/s41591-018-0316-z 30617335

[2] Topol EJ. High-performance medicine: the convergence of human and artificial intelligence. Nat Med. 2019;25(1):44-56. doi:10.1038/s41591-018-0300-7 30617339

[3] Lundervold AS, Lundervold A. An overview of deep learning in medical imaging focusing on MRI. Z Med Phys. 2019;29(2):102-127. doi:10.1016/j.zemedi.2018.11.002 30553609

[4] Titano JJ, Badgeley M, Schefflein J, et al. Automated deep-neural-network surveillance of cranial images for acute neurologic events. Nat Med. 2018;24(9):1337-1341. doi:10.1038/s41591-018-0147-y 30104767

[5] Romano MF, Shih LC, Paschalidis IC, Au R, Kolachalama VB. Large language models in neurology research and future practice. Neurology. 2023;101(23):1058-1067. doi:10.1212/WNL.0000000000207967 37816646 PMC10752640

[6] Maiorana NV, Marceglia S, Treddenti M, et al. Large language models in neurological practice: real-world study. J Med Internet Res. 2025;27:e73212. doi:10.2196/73212 40982758 PMC12453287

[7] Zhou X, Chen Y, Ip FCF, et al. Deep learning-based polygenic risk analysis for Alzheimer's disease prediction. Commun Med (Lond). 2023;3(1):49. doi:10.1038/s43856-023-00269-x 37024668 PMC10079691

[8] Brugnara G, Baumgartner M, Scholze ED, et al. Deep-learning based detection of vessel occlusions on CT-angiography in patients with suspected acute ischemic stroke. Nat Commun. 2023;14(1):4938. doi:10.1038/s41467-023-40564-8 37582829 PMC10427649

[9] Maslej N, Fattorini L, Perrault R, et al. “The AI Index 2025 Annual Report,” AI Index Steering Committee, Institute for Human-Centered AI. Stanford University; 2025.

[10] Artificial Intelligence Degree Program. University of Milan. 2025. Accessed July 2, 2025.unimi.it/it/corsi/facolta-e-scuole/scienze-e-tecnologie/artificial-intelligence

[11] Agenzia per l'Italia Digitale. Italian Strategy for Artificial Intelligence 2024-2026. AGID Website; 2024. Accessed July 4, 2025. agid.gov.it/sites/agid/files/2024_07/Italian_strategy_for_artificial_intelligence_2024-2026.pdf.

[12] Tolentino R, Baradaran A, Gore G, Pluye P, Abbasgholizadeh-Rahimi S. Curriculum frameworks and educational programs in ai for medical students, residents, and practicing physicians: scoping review. JMIR Med Educ. 2024;10:e54793. doi:10.2196/54793 39023999 PMC11294785

[13] Masters K. Artificial intelligence in medical education. Med Teach. 2019;41(9):976-980. doi:10.1080/0142159X.2019.1595557 31007106

[14] Kolachalama VB, Garg PS. Machine learning and medical education. NPJ Digit Med. 2018;1:54. doi:10.1038/s41746-018-0061-1 31304333 PMC6550167

[15] Pinto Dos Santos D, Giese D, Brodehl S, et al. Medical students' attitude towards artificial intelligence: a multicentre survey. Eur Radiol. 2019;29(4):1640-1646. doi:10.1007/s00330-018-5601-1 29980928

[16] Sit C, Srinivasan R, Amlani A, et al. Attitudes and perceptions of UK medical students towards artificial intelligence and radiology: a multicentre survey. Insights Imaging. 2020;11(1):14. doi:10.1186/s13244-019-0830-7 32025951 PMC7002761

[17] Paranjape K, Schinkel M, Nannan Panday R, Car J, Nanayakkara P. Introducing artificial intelligence training in medical education. JMIR Med Educ. 2019;5(2):e16048. doi:10.2196/16048 31793895 PMC6918207

[18] World Economic Forum. Why AI Has a Greater Healthcare Impact in Emerging Markets. World Economic Forum; 2024. Accessed July 4, 2025. weforum.org/stories/2024/06/ai-healthcare-emerging-markets

[19] Voigtlaender S, Pawelczyk J, Geiger M, et al. Artificial intelligence in neurology: opportunities, challenges, and policy implications. J Neurol. 2024;271(5):2258-2273. doi:10.1007/s00415-024-12220-8 38367046 PMC12239762

[20] Grunhut J, Wyatt AT, Marques O. Educating future physicians in artificial intelligence (AI): an integrative review and proposed changes. J Med Educ Curric Dev. 2021;8:23821205211036836. doi:10.1177/23821205211036836 34778562 PMC8580487

[21] Civaner MM, Uncu Y, Bulut F, Chalil EG, Tatli A. Artificial intelligence in medical education: a cross-sectional needs assessment. BMC Med Educ. 2022;22(1):772. doi:10.1186/s12909-022-03852-3 36352431 PMC9646274

[22] Jackson P, Ponath Sukumaran G, Babu C, et al. Artificial intelligence in medical education–perception among medical students. BMC Med Educ. 2024;24(1):804. doi:10.1186/s12909-024-05760-0 39068482 PMC11283685

[23] Ministero dell'Università e della Ricerca. Atti e Normativa. June, 2017. Updated July, 2025. Accessed July 10, 2025. mur.gov.it/it/atti-e-normativa

[24] SurveyMonkey. Sample Size Calculator: Understanding Sample Sizes | SurveyMonkey. SurveyMonkey. 1999. Updated 2025. Accessed July 10, 2025. surveymonkey.com/mp/sample-size-calculator/.

[25] Ardito V, Cappellaro G, Compagni A, Petracca F, Preti LM. Adoption of artificial intelligence applications in clinical practice: insights from a survey of healthcare organizations in lombardy, Italy. Digit Health. 2025;11:20552076251355680. doi:10.1177/20552076251355680 40656847 PMC12254672

[26] Zech J, Pain M, Titano J, et al. Natural language-based machine learning models for the annotation of clinical radiology reports. Radiology. 2018;287(2):570-580. doi:10.1148/radiol.2018171093 29381109

[27] Oh S, Kim JH, Choi SW, Lee HJ, Hong J, Kwon SH. Physician confidence in artificial intelligence: an online Mobile survey. J Med Internet Res. 2019;21(3):e12422. doi:10.2196/12422 30907742 PMC6452288

[28] Allam AH, Eltewacy NK, Alabdallat YJ, et al. Knowledge, attitude, and perception of Arab medical students towards artificial intelligence in medicine and radiology: a multi-national cross-sectional study. Eur Radiol. 2024;34(7):1-14. doi:10.1007/s00330-023-10509-2 PMC11213794 38150076

[29] Chan CKY, Hu W. Students' voices on generative AI: perceptions, benefits, and challenges in higher education. Int J Educ Technol High Educ. 2023;20(1):43. doi:10.1186/s41239-023-00411-8

[30] Davenport T, Kalakota R. The potential for artificial intelligence in healthcare. Future Healthc J. 2019;6(2):94-98. doi:10.7861/futurehosp.6-2-94 PMC6616181 31363513

[31] Rajkomar A, Dean J, Kohane I. Machine learning in medicine. N Engl J Med. 2019;380(14):1347-1358. doi:10.1056/NEJMra1814259 30943338

[32] Ngo B, Nguyen D, Vansonnenberg E. The cases for and against artificial intelligence in the medical school curriculum. Radiol Artif Intell. 2022;4(5):e220074. doi:10.1148/ryai.220074 36204540 PMC9530767

[33] Schubert T, Oosterlinck T, Stevens RD, Maxwell PH, van der Schaar M. AI education for clinicians. EClinicalMedicine 2025;79:102968. doi:10.1016/j.eclinm.2024.102968 39720600 PMC11667627

[34] Rincón EHH, Jimenez D, Aguilar LAC, Flórez JMP, Tapia ÁER, Peñuela CLJ. Mapping the use of artificial intelligence in medical education: a scoping review. BMC Med Educ. 2025;25(1):526. doi:10.1186/s12909-025-07089-8 40221725 PMC11993958

[35] Harrison DS, Chhabra N. Simulation in neurology residency: tools to succeed but still Mountains to overcome. Neurol Educ. 2024;3(4):e200167. doi:10.1212/NE9.0000000000200167 39748897 PMC11694800

[36] Albin CSW, Pergakis MB, Sigman EJ, et al. Education research: junior neurology residents achieve competency but not mastery after a brief acute ischemic stroke simulation course. Neurol Educ. 2023;2(2):e200071. doi:10.1212/ne9.0000000000200071 39469342 PMC11514434

[37] Gigola F, Amato T, Del Riccio M, Raffaele A, Morabito A, Coletta R. Artificial intelligence in clinical practice: a cross-sectional survey of paediatric surgery residents' perspectives. BMJ Health Care Inform. 2025;32(1):e101456. doi:10.1136/bmjhci-2025-101456 PMC12097045 40398897

[38] Lazarus MD, Truong M, Douglas P, Selwyn N. Artificial intelligence and clinical anatomical education: promises and perils. Anat Sci Educ. 2024;17(2):249-262. doi:10.1002/ase.2221 36030525

[39] Morley J, Machado CCV, Burr C, et al. The ethics of AI in health care: a mapping review. Soc Sci Med. 2020;260:113172. doi:10.1016/j.socscimed.2020.113172 32702587

[40] Lekadir K, Frangi AF, Porras AR, et al. FUTURE-AI: international consensus guideline for trustworthy and deployable artificial intelligence in healthcare. doi:10.1136/bmj.r340. BMJ. 2025;388:e081554. doi:10.1136/bmj-2024-081554 39909534 PMC11795397

[41] Chiang S, Picard RW, Chiong W, et al. Guidelines for conducting ethical artificial intelligence research in neurology: a systematic approach for Clinicians and researchers. Neurology. 2021;97(13):632-640. doi:10.1212/WNL.0000000000012570 34315785 PMC8480407

